# A robust cross-crop disease detection framework based on SIS-YOLOv11 with climate-adaptive mechanisms

**DOI:** 10.1371/journal.pone.0353863

**Published:** 2026-07-17

**Authors:** Yiming Wang, Ruiqian Qin, Zhuo Zhang

**Affiliations:** 1 Changchun Institute of Education, Changchun, Jilin, China; 2 College of Information Technology, Jilin Agricultural University, Changchun, Jilin, China; 3 Changchun Polytechnic University, Changchun, Jilin, China; Southern Federal University Academy of Biology and Biotechnology named after D I Ivanovsky: Uznyj federal’nyj universitet Akademia biologii i biotehnologii im D I Ivanovskogo, RUSSIAN FEDERATION

## Abstract

Plant disease detection under complex climatic conditions and cross-crop scenarios remains a critical challenge. To address this, we propose a novel SimAM-Inception-StyleRandomization (SIS)-YOLOv11 algorithm based on YOLOv11n for early/late blight detection on potato and tomato leaves. Our core innovations are: 1) A C3k2-SSI module integrating Style Randomization, Inception architecture, and SimAM attention to enhance cross-crop generalization; 2) A Fusion-InceptionConv module for fine-grained feature extraction under rainfall/haze noise; 3) SPPF-Inception and C2PSA-IS modules to optimize multi-scale feature fusion; 4) DepGraph pruning to reduce 47.82% parameters while improving performance. Experiments show that the pruned SIS-YOLOv11 outperforms YOLOv11n by 3.7% in precision, 6.6% in recall, 5.4% in mAP50, and 7.9% in mAP50-95, and surpasses mainstream models (Faster R-CNN, SSD, etc.). This study provides a robust, lightweight solution for automated cross-crop disease detection in complex agricultural environments.

## Introduction

Potato and tomato, key Solanaceae crops, play an indispensable role in global agriculture owing to their extensive cultivation, substantial economic impact, and vital nutritional value. Real-time crop monitoring and disease diagnosis are crucial for safeguarding growth and improving yield in modern precision agriculture. Early blight and late blight [1–6], two prevalent foliar diseases affecting these crops, severely impair growth, reduce yield, and cause significant economic losses. Thus, developing timely and accurate identification technologies for these diseases is of great practical significance for advancing precision agricultural management and enhancing crop productivity [7–12].

Traditional disease monitoring relies heavily on manual inspection, which is labor-intensive, time-consuming, and prone to errors due to subjective judgment and individual variability. These limitations render manual methods increasingly inadequate for large-scale crop disease surveillance [13–18].

The rapid advancement of artificial intelligence, particularly deep learning in image recognition, has revolutionized crop disease monitoring by enabling automatic feature extraction and detection from large image datasets. This technology significantly improves the efficiency and accuracy of disease diagnosis, providing robust technical support for smart agriculture [19–24]. Among deep learning-based object detectors, the YOLO (You Only Look Once) series is particularly promising for real-time applications, as it accomplishes detection in a single inference pass, balancing high speed and accuracy—making it ideal for large-scale, real-time crop disease monitoring. Continuous optimizations of YOLO models have yielded excellent performance in crop disease identification, further promoting the development of intelligent agriculture.

Numerous studies have validated the effectiveness of improved deep learning models for crop pest and disease detection. Zhang et al. [25] proposed an enhanced YOLOX-based model integrated with Efficient Channel Attention, hard-Swish activation, and Focal Loss, which improved feature extraction, mitigated class imbalance, and enhanced the accuracy and speed of cotton pest and disease detection. Xiong et al. [26] developed the YOLOv5s_HSSE model for automatic detection and counting of two major kiwi pests on sticky traps, addressing the high cost and inaccuracy of conventional monitoring methods. Luo et al. [27] proposed a lightweight Light-SA YOLOv8 model to enhance real-time citrus pest and disease detection under complex orchard conditions, tackling challenges in small-target detection and cluttered backgrounds. Soeb et al. [28] developed a YOLOv7-based tea disease detection method with data augmentation to address sample scarcity, achieving high-precision results that outperformed existing algorithms and providing an effective solution for rapid tea disease identification.

Despite these advances, deep learning models still face notable challenges in real-world agricultural environments. First, agricultural images captured under complex climatic conditions (e.g., rain, haze) or cluttered backgrounds contain significant noise, which degrades model robustness and generalization. Second, acquiring and annotating crop disease data is resource-intensive, as data for diverse crop diseases are often scarce and require substantial manpower and material resources. Third, mainstream models primarily focus on improving detection accuracy for single-crop diseases, neglecting cross-crop recognition of the same diseases.

To address these gaps, this study proposes an enhanced YOLOv11n-based object detection algorithm, SimAM-Inception-StyleRandomization (SIS)-YOLOv11 (the abbreviation SIS corresponds to the three key innovative technologies of the model: SimAM attention, Inception architecture, and Style Randomization), for early and late blight detection on potato and tomato leaves under complex backgrounds. Key innovations include: (1) A C3k2-SSI layer designed to enhance cross-crop generalization for the same diseases; (2) Integration of the Fusion-InceptionConv [29] structure to replace YOLOv11n’s convolutional layers, improving fine-grained feature extraction under noisy conditions (e.g., rain, haze) by capturing detailed textural characteristics of diseased leaves; (3) An SPPF-Inception layer to facilitate multi-scale feature fusion in the feature pyramid, decoupling critical features from the channel dimension to enhance robustness and generalization; (4) A C2PSA-IS layer with multi-scale attention for efficient feature fusion; (5) Application of the DepGraph [30] pruning method, reducing model parameters by 47.82% while achieving higher accuracy than the original YOLOv11n.

## Materials and methods

### Dataset construction

#### Image acquisition.

**Fig 1:**
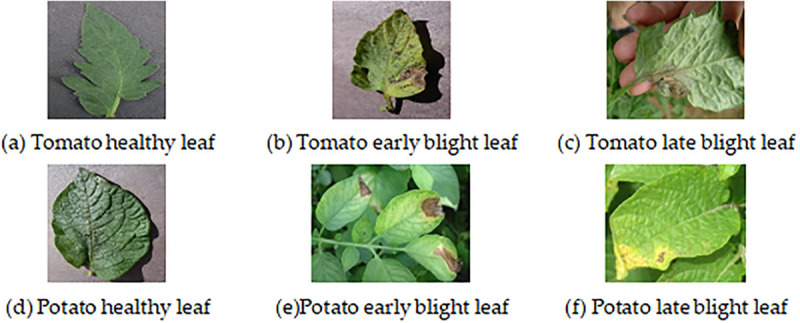
A subset of dataset images showcasing different plant leaf types.

This study curated images of potato and tomato leaves affected by early blight, late blight, and healthy conditions from the public PlantVillage dataset, captured under laboratory conditions. Additionally, web crawling techniques were employed to collect images of potato and tomato leaves with early blight, late blight, and healthy conditions from field environments available on the internet. These images were integrated to create a comprehensive dataset for early blight, late blight, and healthy conditions of potato and tomato leaves. The dataset comprises 3,268 images, with 1,628 images of potato leaves and 1,640 images of tomato leaves. A subset of the images used in this experiment is presented in Fig 1.

During the data annotation phase, this study utilized LabelImg software to annotate images of potato and tomato leaves exhibiting early blight, late blight, and healthy conditions, which were either selected from the PlantVillage public dataset or collected from the internet via web crawling techniques.

#### Data augmentation.

**Fig 2:**
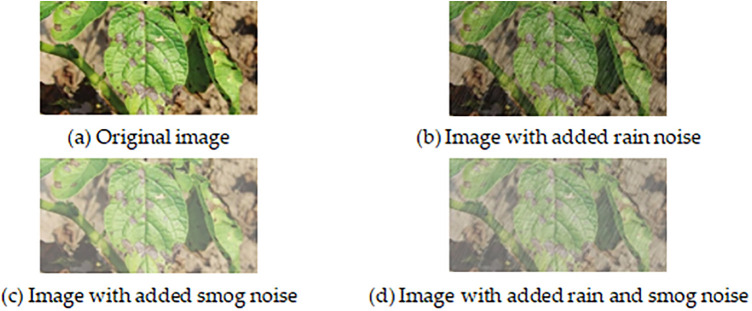
An example of added climate noise.

In this study, to enhance the generalization ability and robustness of the model, multiple data augmentation strategies were applied to preprocess the original dataset. Initially, a program was developed using the Python programming language and the OpenCV library to incorporate simulated rainfall and haze as complex background conditions. This program was employed to augment the dataset by randomly introducing simulated agricultural climate noise, including rainfall and haze, to the images. By integrating such agricultural climate noise, these augmentations not only enriched the diversity of the dataset but also improved the model’s robustness in recognizing plant diseases under complex climatic conditions. The simulated agricultural climate rainfall and haze noise introduced in this study are illustrated in Fig 2.

Furthermore, this study applied various image augmentation techniques to the dataset, including stretching, scaling, and HSV color space transformation. These methods effectively expanded the dataset’s size while increasing the diversity and complexity of the samples. Stretching and scaling operations enabled the model to better adapt to disease features at different scales, whereas HSV color space transformation enhanced the model’s robustness to variations in illumination.

#### Production of datasets.

In this study, the augmented dataset was utilized, with the potato disease dataset designated as the training set and the tomato disease dataset as the validation set. The training set comprised 785 images of tomato leaves affected by early blight, 805 images of healthy tomato leaves, and 850 images of tomato leaves affected by late blight. The validation set included 831 images of potato leaves affected by early blight, 800 images of healthy potato leaves, and 827 images of potato leaves affected by late blight.

### Algorithm improvement and pruning

#### YOLOv11 network model.

YOLOv11 (You Only Look Once version 11) represents the latest iteration of the YOLO series of object detection algorithms. Compared to its predecessors, YOLOv11 offers improved detection accuracy while maintaining a reduced number of model parameters. Since its inception, the YOLO series has focused on real-time object detection, with its core principle being the simultaneous execution of image classification and regression tasks through a single neural network architecture.

#### Improved YOLOv11 algorithm: SIS-YOLOv11.

**Fig 3:**
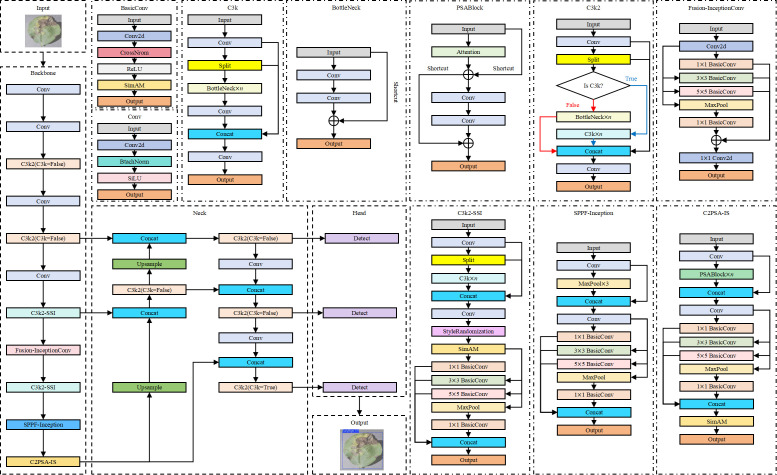
Structure diagram of SIS-YOLOv11 target detection algorithm.

Although the YOLOv11 object detection algorithm has achieved excellent performance across various object detection tasks, its detection accuracy for plant diseases under complex climatic conditions remains limited, with suboptimal feature extraction capabilities and poor domain generalization across different crops affected by the same disease. To address these challenges, this study proposes an improved YOLOv11 algorithm, introducing a novel SIS-YOLOv11 algorithm designed for cross-crop disease detection in complex climatic backgrounds. The model architecture of the proposed algorithm is illustrated in Fig 3.

#### SimAM attention mechanism.

To enhance the model’s ability to distinguish between important and unimportant features and improve its feature extraction performance in complex agricultural backgrounds, this study incorporates the SimAM attention mechanism [31]. Drawing on neuroscience principles, SimAM evaluates the importance of neurons by assessing their linear separability, thereby enhancing meaningful features in the feature map while suppressing irrelevant ones. This mechanism mimics the variability in neuronal firing patterns and their mutual inhibition in the spatial domain, increasing the model’s focus on critical regions. The neuron competence function of SimAM is given by Eq (1), which mathematically quantifies the linear separability of neurons to determine their importance weights in the feature extraction process.


$$\begin{array}{c} e_{t}^{*}=\frac{4\left(\hat{\sigma^{2}}+\lambda \right)}{(t-\hat{\mu }{)}^{2}+2{\hat{\sigma }}^{2}+2\lambda } \end{array}$$ (1)

Here, μ represents the mean of the input feature map across the *H* and *W* dimensions, and σ denotes the standard deviation of the input feature map across the same dimensions. The parameter λ typically serves as a scaling factor in the context, controlling the response intensity of the energy function to the features. A lower energy value indicates a greater distinction between a neuron and its surrounding neurons, signifying higher importance. Consequently, the importance of a neuron can be derived as $1/e^{*}$.

#### Style randomization.

To enhance the model’s robustness to unseen domains (e.g., shared disease features across potato and tomato leaves), this study introduces Style Randomization [32] to design and implement the C3k2-SSI module. This approach generates new features with randomized styles by transforming the features of original samples. To achieve this, an encoder-decoder network is employed to integrate noise information with the original style information in the latent space. Compared to traditional image-based data augmentation methods, this method is more targeted and effective for addressing domain generalization challenges.

To achieve the above-mentioned Style Randomization mechanism, an encoder-decoder network is employed in each sub-module to integrate domain-specific noise information with the original style information of the corresponding source domain in the latent space. The parameter *K* directly determines the number of such domain-specific sub-modules: when *K* = 0, no source domain is involved and Style Randomization is not performed; as *K* increases, more source domain-specific sub-modules are activated, enabling the model to learn style variations across multiple crop domains. Notably, when *K* > the actual number of source domains in the dataset, redundant sub-modules may lead to overfitting; when *K* < the actual number of source domains, partial crop domain features cannot be fully stylized, thus weakening the generalization ability. This adjustable *K* design allows flexible adaptation to different cross-crop detection tasks by matching the number of source domains, which is a key advantage over fixed-style augmentation methods.

**Fig 4:**
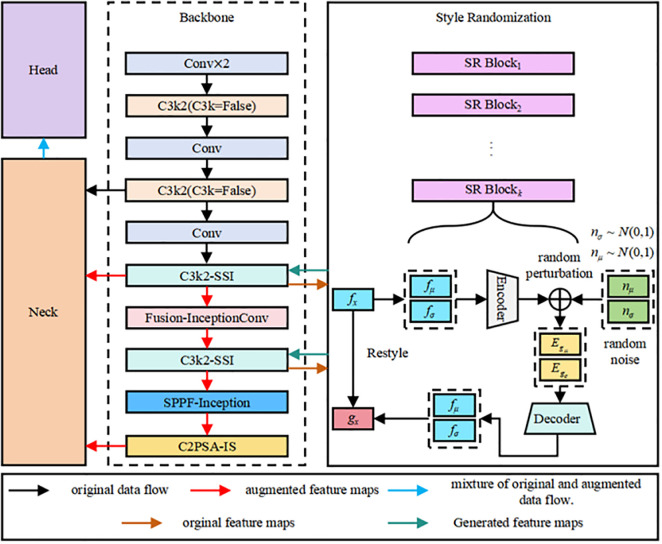
Function diagram of style randomization. $f_{x}$ is the original feature map, $g_{x}$ is the characteristic diagram after increasing the noise, μ is the channel mean value of $f_{x}$ or $g_{x}$, and σ is the channel standard deviation of $f_{x}$ or $g_{x}$.

The role of Style Randomization within the SIS-YOLOv11 network architecture is illustrated in Fig 4. By randomly adding noise to the feature map for style transformation, the proposed module operates in the feature space, enabling diverse and abstract transformations of input images. Consequently, compared to image-based augmentation, the enhanced features are expected to cover a broader range of possible styles or distributions.

As depicted in Fig 3, this study introduced a novel convolutional structure named BasicConv2D. The BasicConv2D structure incorporates a standard 2D convolutional layer (Conv-2D) for preliminary feature extraction and enhances the model’s generalization capability through the introduction of the CrossNorm method [33], which demonstrates notable performance on cross-domain data. CrossNorm improves generalization to feature maps of different styles by exchanging the mean and standard deviation across channels of paired feature maps. Subsequently, a ReLU activation function is applied to introduce non-linear characteristics, further enhancing the model’s expressive capacity. Finally, the SimAM attention mechanism strengthens important features in both channel and spatial dimensions while suppressing irrelevant ones, thereby improving the model’s contextual understanding and robustness for disease detection tasks under agricultural climatic conditions (e.g., fog and rainfall). Through this structural design, BasicConv2D not only achieves efficient feature extraction but also leverages CrossNorm and SimAM to further enhance the model’s generalization and robustness.

#### Fusion-InceptionConv.

As illustrated in Fig 3. this study introduces the Fusion-InceptionConv module to replace the convolutional layers in YOLOv11n, enhancing the model’s robustness in feature extraction under rainy and foggy backgrounds. This module is an improvement over the Conv module of YOLOv11n and the InceptionV1 architecture [34], comprising a 3x3 convolutional layer and multiple Inception branches. The latter capture multi-scale features through parallel 1x1, 3x3, and 5x5 convolutional layers and a pooling layer. The outputs from all paths are subsequently fused using a 1x1 convolutional layer. The Fusion-InceptionConv module replaces the concatenation operation of InceptionV1 with an addition operation, optimizing feature fusion and improving the model’s generalization and robustness in complex climatic conditions.

#### C3k2-SSI.

As shown in Fig 3, this study proposes an enhanced C3k2 module, named C3k2-SSI, for the original YOLOv11n by integrating Style Randomization, the InceptionV1 architecture, and the SimAM attention mechanism. The C3k2-SSI module builds upon the original C3k2 module of YOLOv11n by incorporating Style Randomization to perform random stylization of feature maps, thereby improving the model’s domain generalization and robustness in extracting features of the same disease across different crops. Additionally, a modified InceptionV1 structure with four branches is employed to enhance the model’s ability to decouple fine-grained leaf disease features. Finally, the SimAM attention mechanism is integrated to suppress irrelevant features while preserving meaningful ones.

#### SPPF-Inception.

As illustrated in Fig 3, this study proposes an enhanced SPPF module for YOLOv11n, named SPPF-Inception, based on the InceptionV1 architecture. The SPPF-Inception module adopts the concept of feature pyramid networks, enabling further multi-scale feature fusion.

#### C2PSA-IS.

As depicted in Fig 3, this study proposes an enhanced C2PSA module, named C2PSA-IS (C2PSA-Inception-SimAM), to improve feature extraction capability and robustness under complex climatic backgrounds. This module builds upon the original C2PSA module of YOLOv11n with innovative improvements, integrating a modified InceptionV1 architecture and the SimAM attention mechanism. The C2PSA-IS module effectively handles diverse disease texture features and background noise in agricultural environments. The original C2PSA module incorporates a Pyramid Slicing Attention (PSA) mechanism, which enhances the model’s focus on fine-grained features through multi-level feature weighting. However, for diverse disease datasets in agricultural climates, particularly under challenges posed by noise interference (e.g., fog and rainfall), the original C2PSA module exhibits certain limitations in complex backgrounds. To address this, C2PSA-IS innovatively incorporates a modified Inception structure and the SimAM attention mechanism to enhance the model’s feature decoupling capability, fine-grained feature extraction, and multi-scale feature fusion performance.

#### DepGraph pruning.

DepGraph is a versatile automated structured pruning method designed to achieve efficient pruning across various neural network architectures, including CNNs, Transformers, RNNs, and GNNs. This method analyzes the complex inter-layer dependencies within the network to automatically group coupled parameters. By recursively deriving a grouping matrix G based on local dependencies between adjacent layers, DepGraph prunes redundant parameters within the same group, thereby reducing model complexity. Local dependencies between adjacent layers are modeled using a Dependency Graph, which simplifies the problem to a path search task. If a path exists between nodes in the dependency graph, they are assigned to the same group. This approach effectively identifies and manages inter-layer dependencies in the SIS-YOLOv11 model, enabling the pruning of redundant parameters and significantly enhancing model performance.

Notably, the pruning operation is performed prior to sparse training, with the aim of reducing model redundancy and establishing a foundation for subsequent sparse optimization. The fine-tuning process is then conducted exclusively on the pruned model, which has undergone lightweight structural adjustment, to further refine its performance through targeted parameter optimization.

Pruning the trained model involves two primary steps:

1. Sparse Training: This technique introduces sparsity during the training process by directly setting certain network weights to zero, thereby simplifying the model structure. Sparsification is typically achieved using methods such as L1 regularization and pruning masks, which progressively reduce the number of redundant parameters to lower computational complexity and storage requirements. This process enables the network to maintain high expressive capacity with reduced computational resources.
2. Fine-Tuning: Following pruning or sparsification, fine-tuning optimizes the remaining parameters through small-step updates. This step mitigates performance degradation caused by pruning, ensuring that the pruned model’s performance closely matches or even surpasses that of the original model. Fine-tuning typically employs a smaller learning rate to meticulously adjust network weights, preserving the effective features learned during training.Fine-tuning is conducted on the pruned model architecture, which has been pre-optimized through DepGraph pruning, allowing for enhanced feature extraction efficiency and performance improvement despite parameter reduction.

## Results

### Performance metrics for network models

This study employs Precision (P), Recall (R), and mean Average Precision (mAP) as evaluation metrics for the experiments. The formulas for these metrics are presented as Eq (2), Eq (3), Eq (4) and, Eq (5).


$$\begin{array}{c} \text{ Precision }=\frac{TP}{TP+FP}\times 100\% \end{array}$$ (2)

$$\begin{array}{c} \text{ Recall }=\frac{TP}{TP+FN}\times 100\% \end{array}$$ (3)

$$\begin{array}{c} AP=\int_{0}^{1}P(R)dR\times 100\% \end{array}$$ (4)

$$\begin{array}{c} mAP=\frac{\sum_{1}^{N}\int_{0}^{1}P(R)dR}{N}\times 100\% \end{array}$$ (5)

True Positive *TP* is defined as a detection result where the Intersection over Union (IOU) between the predicted bounding box and the manually annotated bounding box is ⩾0.5 *consistent with the COCO* 2017 *evaluation standard*, and the disease category is correctly identified;

Where:

False Positive *FP* refers to a detection result where the IOU < 0.5 or the disease category is misclassified;

False Negative *FN* denotes a diseased region in the image that is not detected by the model;

AP *Average Precision* is the area under the Precision-Recall P−R curve for a single disease category, and mAP is the average of AP values across all categories *healthy leaves*, *early blight*, *late blight*;

mAP50 refers to mAP calculated with an IOU threshold of 0.5, and mAP50-95 represents the average of mAP values calculated with IOU thresholds ranging from 0.5 to 0.95 *stepping by* 0.05, which comprehensively evaluates the model’s detection performance under different overlap requirements.

### Experiment environments

The experiments were conducted using the PyTorch deep learning framework on a Windows 10 Professional system. The hardware configuration included a 12th Gen Intel® Core™ i9-12900HX CPU operating at 2.30 GHz, an NVIDIA GeForce RTX 3080Ti Laptop GPU, and 64 GB of RAM. The experimental programs were developed using CUDA 11.8 and Python 3.9.

The tomato disease dataset was utilized as the training set, while the potato disease dataset served as the validation set. The training set comprised 1,648 samples, and the validation set contained 1,628 samples, resulting in a 1:1 ratio between the training and validation sets. In this study, identical initial training parameters were set for each experiment. The input image resolution was 640×640 pixels, and the training was conducted for 70 epochs. All other parameters adhered to the default settings of YOLOv11n.

To ensure the fairness and reliability of comparative experiments, all models involved in the comparison were trained and tested under identical experimental parameters and environmental configurations.

### Experimental results of k-value parameter of style randomization

This experiment investigates the impact of the *K* value in Style Randomization on model accuracy, with *K* ranging from 1 to 10. The results are presented in Table 1. The experimental findings indicate that when *K* = 1, the model achieves the best overall performance across multiple evaluation metrics. Even with *K* = 1, Style Randomization can effectively alter the original style information by introducing random noise, thereby generating new features with randomized styles.

**Table 1:** K-value experiment of style randomization.

K value	Precision	Recall	mAP50	mAP50-95
1	84.3%	79.6%	84.8%	80.8%
2	78.9%	72.3%	79.4%	75.7%
3	73.8%	76.8%	79.1%	74.9%
4	79.5%	78.0%	83.3%	78.2%
5	79.1%	75.7%	82.0%	76.8%
6	76.1%	77.9%	80.9%	77.6%
7	80.2%	75.9%	81.5%	77.2%
8	80.6%	76.5%	81.2%	77.2%
9	73.0%	70.6%	76.9%	73.0%
10	81.8%	77.7%	83.8%	78.6%

### Ablation experiments

The results of the ablation experiments are presented in Table 2. When the C3k2-SSI, Fusion-InceptionConv, SPPF-Inception, and C2PSA-IS modules were individually introduced, the model’s accuracy decreased, indicating that these four modules must be combined to enhance model performance. For the C3k2-SSI module, its contribution was more pronounced when integrated with other modules, particularly in the full-module combination, where the model achieved an accuracy of 84.3%. This suggests that the C3k2-SSI module plays a critical role in driving the overall performance of the model.

**Table 2:** Experimental results of SIS-YOLOv11 algorithm ablation.

Network Settings				Object detection algorithm evaluation index				Parameter Quantity
a	b	c	d	Precision	Recall	mAP50	mAP50-95	Params· M
×	×	×	×	82.3%	74.9%	82.8%	76.8%	2.58
✓	×	×	×	77.4%	74.3%	79.9%	75.5%	3.32
×	✓	×	×	80.3%	73.6%	81.2%	78.0%	5.01
×	×	✓	×	77.9%	73.9%	79.9%	76.4%	3.17
×	×	×	✓	72.6%	76.1%	79.2%	74.0%	3.17
✓	✓	×	×	83.0%	78.3%	84.2%	80.1%	5.74
×	✓	✓	×	75.4%	76.8%	78.5%	73.6%	5.60
×	×	✓	✓	81.0%	76.0%	83.3%	78.8%	3.76
✓	×	×	✓	77.3%	74.6%	79.0%	75.4%	3.91
✓	✓	✓	×	71.9%	76.0%	76.2%	72.6%	6.33
×	✓	✓	✓	73.6%	72.7%	74.7%	69.9%	6.19
✓	×	✓	✓	78.9%	77.8%	83.5%	78.3%	4.50
✓	✓	×	✓	81.6%	76.4%	83.1%	77.8%	6.33
✓	✓	✓	✓	84.3%	79.6%	84.8%	80.8%	6.92

Note: × indicates that the improvement strategy is not used, ✓ indicates that the improvement strategy is use. Here, a corresponds to the C3k2-SSI Module, b corresponds to the Fusion-InceptionConv Module, c corresponds to the SPPF-Inception, and d corresponds to the C2PSA-IS.

The Fusion-InceptionConv module enhances the model’s robustness under complex climatic conditions by integrating multi-scale convolutional features. Experimental results show that when introduced alone, the module led to a slight decrease in accuracy (80.3%), with limited performance gains compared to other modules. However, when combined with the C3k2-SSI module, both recall and mean Average Precision (mAP) improved significantly, highlighting its importance in enhancing the model’s ability to process features in complex backgrounds.

The SPPF-Inception module, built upon the InceptionV1 architecture and inspired by Feature Pyramid Networks, further strengthens the model’s capability for multi-scale feature fusion. Although its standalone performance was reduced, with an accuracy of 77.9%, its integration with other modules, particularly the Fusion-InceptionConv module, resulted in an mAP50-95 of 81.0%. This indicates its significant role in improving feature fusion and multi-scale information processing.

The C2PSA-IS module, an improved version of the original C2PSA module, exhibited an accuracy of 72.6% when used independently, substantially lower than other module combinations. However, in combination scenarios, particularly with the full-module setup, the C2PSA-IS module significantly enhanced recall and mAP, demonstrating its improved capability to handle diverse pest and disease texture features and background noise.

The combination of these modules exhibited complementary effects, with the full-module configuration achieving the best performance across all metrics. This underscores that the synergistic interaction of these modules effectively enhances the model’s generalization, feature extraction, and robustness for pest and disease identification in complex agricultural environments. Compared to the baseline YOLOv11n model, the combined module configuration improved accuracy by 2%, recall by 4.7%, mAP50 by 2%, and mAP50-95 by 4%. However, this improvement came with an increase in model parameters by 4.34M. To address this, the study will subsequently apply the DepGraph pruning method to reduce parameter count while maintaining or improving accuracy.

### Pruning experiments

This study set the model pruning rate to 0.5, effectively reducing the model parameters by half. Following sparse training with 500 epochs, the model underwent fine-tuning training for 70 epochs. The performance metrics of the pruned SIS-YOLOv11 model on the dataset used in this study are presented in Table 3. The experimental results demonstrate that after model pruning, the SIS-YOLOv11 model achieved a 1.7% increase in accuracy, a 1.9% improvement in recall, a 3.4% enhancement in mAP50, and a 3.9% increase in mAP50-95 compared to the pre-pruned model, while reducing the parameter count by 3.3M.

**Table 3:** Model pruning experiments.

Stage	Precision	Recall	mAP50	mAP50-95	Params·M
After training	84.3%	79.6%	84.8%	80.8%	6.92
Sparse training	23.2%	65.6%	21.9%	6.3%	3.62
Finetune training	86.0%	81.5%	88.2%	84.7%	3.62

### Comparison experiments

The proposed SIS-YOLOv11 model was compared with FasterRCNN, SSD, YOLOv3-tiny, YOLOv5n, YOLOv8n, and YOLOv11n, with the results presented in Table 4. FasterRCNN, a two-stage object detection algorithm, exhibits lower detection efficiency. The comparative experimental results demonstrate that the pruned SIS-YOLOv11 model outperforms all other models across all metrics, except for a 1.04M increase in parameter count compared to YOLOv11n. Relative to YOLOv11n, the pruned SIS-YOLOv11 model achieves a 3.7% improvement in accuracy, a 6.6% increase in recall, a 5.4% enhancement in mAP50, and a 7.9% improvement in mAP50-95.

**Table 4:** Model comparison experiments.

Model	Precision	Recall	mAP50	mAP50-95	Params·M
SSD	75.9%	48.9%	59.9%	38.2%	26.30
FasterRCNN	57.5%	46.9%	56.4%	36.5%	137.10
YOLOv3-tiny	78.0%	74.9%	80.7%	77.1%	103.67
YOLOv5n	76.0%	75.4%	78.8%	68.9%	1.76
YOLOv8n	77.5%	78.2%	81.6%	77.7%	3.01
YOLOv11n	82.3%	74.9%	82.8%	76.8%	2.58
SIS-YOLOv11	84.3%	79.6%	84.8%	80.8%	6.92
SIS-YOLOv11(pruned)	86.0%	81.5%	88.2%	84.7%	3.62

**Fig 5:**
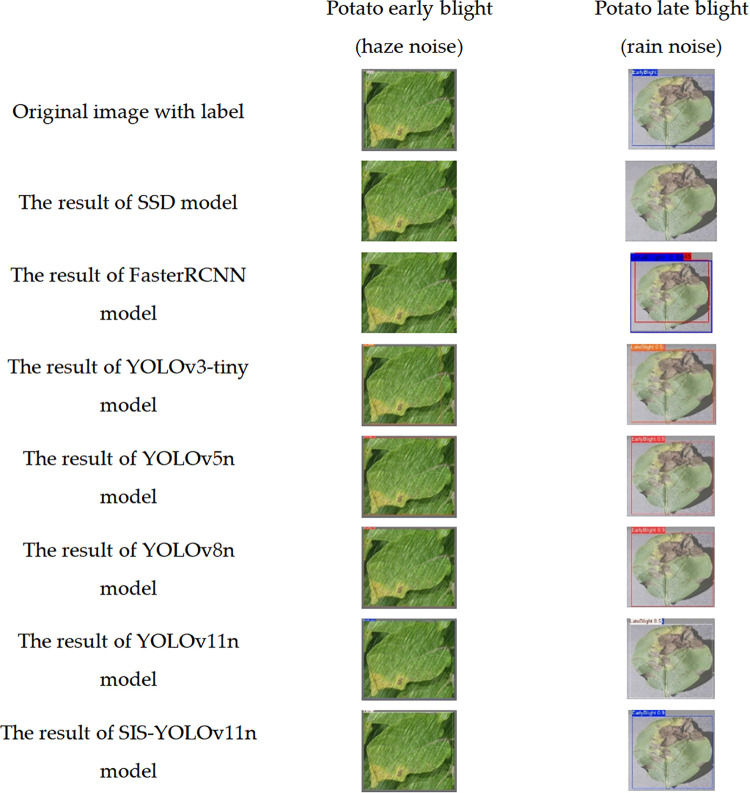
Comparison of detection results.

The detection results of the comparative experiments are shown in Fig 5. The results indicate that the SIS-YOLOv11 model accurately detects plant diseases under both haze and rainfall noise conditions. In contrast, other models either failed to generate detection boxes or produced incorrect detections under these noise conditions.

## Discussion

**Fig 6:**
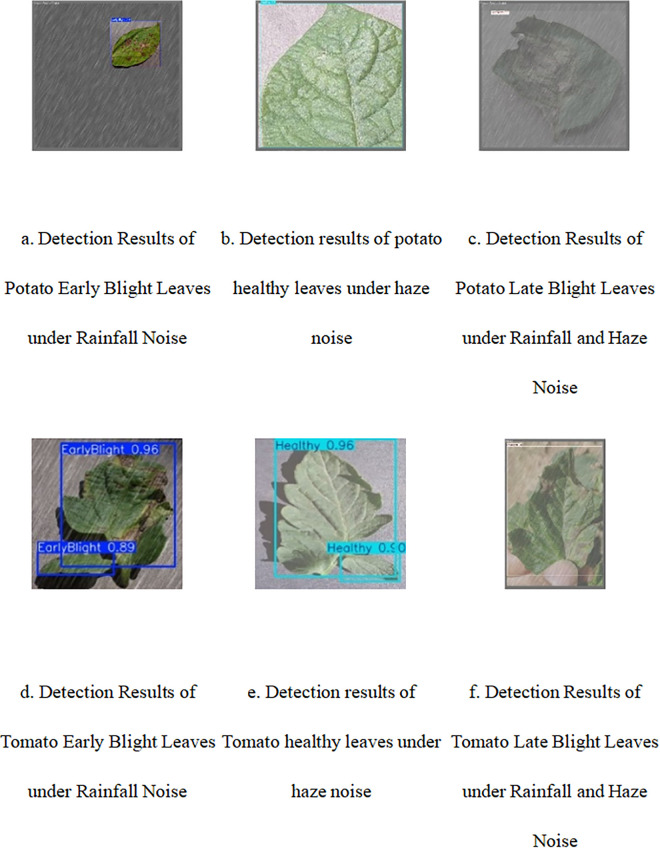
SIS-YOLOv11 algorithm detection results.

The detection results of the SIS-YOLOv11 model on simulated rainfall and haze noise conditions for potato and tomato disease leaf image samples are presented in Fig 6. The results demonstrate that the SIS-YOLOv11 model exhibits robustness in complex backgrounds. This indicates that the incorporated C3k2-SSI module performs effectively in cross-domain experiments, where the tomato disease dataset was used as the training set and the potato disease dataset as the validation set, enhancing the model’s robustness and generalization capability. The Fusion-InceptionConv module enables the decoupling of finer-grained disease features, improving the model’s feature extraction capability and accuracy under complex agricultural backgrounds, such as afternoon lighting and haze conditions. The SPPF-Inception module facilitates feature fusion following the original feature pyramid structure, while the C2PSA-IS module further enhances feature integration. The synergistic integration of these four modules significantly improves the model’s performance in cross-crop disease detection tasks under challenging climatic conditions.

Significantly, the SIS-YOLOv11 model surpasses the YOLOv11n baseline by 1.7 percentage points in precision, 1.9 percentage points in recall, and 3.4 percentage points in mAP. These metrics highlight the model’s enhanced discriminatory power and reliability in accurately classifying diseases across heterogeneous crops and inclement environmental conditions. The observed performance gains are particularly pronounced in scenarios fraught with challenging illumination dynamics and weather-induced interferences, substantiating the model’s robustness against real-world perturbations. Moreover, its demonstrated ability to generalize effectively to novel crop diseases underscores its translational potential for broad-scale deployment in diverse agricultural ecosystems.

These findings collectively underscore the SIS-YOLOv11 model’s technical superiority and practical relevance for precision agriculture. By offering improved detection fidelity and environmental resilience, the model holds promise to revolutionize disease management strategies, potentially mitigating crop losses and optimizing yield outcomes. Its advancements in feature disentanglement and multimodal integration establish a compelling foundation for future research in developing intelligent diagnostics systems for sustainable agriculture.

## Conclusion

This study addresses the challenges of complex climatic conditions (rainfall and haze) and cross-crop scenarios (training on tomato samples and validating on potato samples) by proposing a novel algorithm, SIS-YOLOv11, for cross-crop disease detection in challenging weather conditions. The key findings and future directions are summarized as follows:

1. Model Enhancements: The C3k2-SSI module was designed to enhance the model’s generalization capability across different crop diseases. The Fusion-InceptionConv module was introduced to extract fine-grained features, while the SPPF-Inception module was developed for multi-scale feature fusion. Additionally, the C2PSA-IS module was designed to improve the model’s ability to extract disease-specific features, thereby enhancing robustness under complex climatic noise.
2. Model Optimization: To eliminate redundant parameters and improve performance, the DepGraph method was employed for model pruning. The optimized network structure resulted in enhanced model performance.
3. Limitations and Future Work: This study primarily focused on early and late blight diseases in potato and tomato crops. Future research could extend to other crops and diseases. Additionally, the dataset size in this study was relatively small, and collecting more diverse data could further improve the model’s generalization capability.

The core contribution of this work lies in the development of the SIS-YOLOv11 framework, which innovatively integrates the C3k2-SSI, Fusion-InceptionConv, SPPF-Inception, and C2PSA-IS modules to address cross-crop generalization and complex climatic interference challenges, while DepGraph pruning achieves a 47.82% parameter reduction without performance loss. The pruned model outperforms YOLOv11n by 3.7% in precision, 6.6% in recall, 5.4% in mAP50, and 7.9% in mAP50-95, and surpasses mainstream models such as Faster R-CNN and SSD, providing a robust, lightweight end-to-end solution for cross-crop disease detection in practical agricultural environments.
