# Supplementary material for: A robust cross-crop disease detection framework based on SIS-YOLOv11 with climate-adaptive mechanisms
Source: PLoS One. 2026 Jul 17;21(7):e0353863. doi: 10.1371/journal.pone.0353863 (PMC13378986; doi:10.1371/journal.pone.0353863)
Supplement: Attachment — Submitted filename: Response to Reviewers.pdf [file pone.0353863.s002.pdf]

Response to Reviewers

Dear Editors and Reviewers,

We sincerely appreciate the valuable comments and constructive suggestions provided by all reviewers. Their insights have greatly helped us improve the quality and completeness of this manuscript. We have carefully addressed each comment and made corresponding revisions to the paper. All modified content in the revised manuscript has been marked in red for the convenience of reviewers to quickly identify. Below is a detailed response to each reviewer's feedback.

Response to Reviewer #1

Thank you very much for your positive evaluation and recognition of our work. We greatly appreciate your affirmation of the novelty of the SIS-YOLOv11 framework, architectural innovations, and experimental validity.

Response to Reviewer #2

Comment: 1. The content in the introduction needs improvement.

Response:

Thank you for your valuable suggestion. We have comprehensively revised the entire Introduction section to enhance its academic rigor, logical coherence, and readability. Specifically, we optimized the overall structure of the background narrative, supplemented the latest advances in cross-crop disease detection and climate-adaptive modeling, strengthened the comparative analysis between our work and existing literature to highlight the novelty of the proposed framework, and refined the language expression by simplifying cumbersome sentences. The fully revised Introduction section is presented in the revised manuscript.

Comment: 2. To enhance the model' s generalization and robustness, the authors introduced and modified the Style Randomization module. However, the role of the parameter K within this module is not explained, even though its effect on model accuracy is discussed later. Based on the paper, can K be considered as the weight for randomizing the original data, with K = 1 yielding the best performance on the original samples? If so, what is the significance of analyzing K in the subsequent discussions?

Response:

We thank the reviewer for this valuable comment. We have added detailed

explanations of parameter K in the Style Randomization module section of the revised paper. K denotes the number of domain-specific sub-modules in the module: K=0 represents no style randomization; increasing K enables the model to learn style variations across more crop domains, while an unsuitable K (either too large or too small) will lead to overfitting or insufficient generalization respectively. The adjustable K allows flexible adaptation to different cross-crop detection tasks, which is a core merit of our module. The subsequent analysis of K is thus to quantitatively validate the effect of this parameter on the model's generalization and robustness, and further confirm the superiority of our adjustable design.

Comment: 3. In the pruning experiments, the authors did not specify when the pruning operation was performed—was it done before sparse training or before fine-tuning? After pruning, the model's parameter count decreased by 3.3M, yet all performance metrics improved. Why did the model's performance increase despite the reduction in parameters? Please provide clarification in the paper.

Response:

We appreciate your observation and supplemented details in the DepGraph Pruning section: (1) Pruning is implemented prior to sparse training to eliminate redundant structures; (2) Fine-tuning is exclusively conducted on the pruned lightweight model. Performance improvement after parameter reduction is attributed to pruning-removed feature interference and fine-tuning-optimized parameter distribution, achieving both lightweighting and performance enhancement.

Comment: 4. The Discussion section is brief and lacks sufficient depth. The authors are encouraged to expand this section by providing a more thorough analysis of the experimental results, such as explaining why the model's performance improved after pruning.

Response:

We appreciate your observation and supplemented details in the DepGraph Pruning section: (1) Pruning is implemented prior to sparse training to eliminate redundant structures; (2) Fine-tuning is exclusively conducted on the pruned lightweight model. Performance improvement after parameter reduction is attributed to pruning-removed feature interference and fine-tuning-optimized parameter distribution, achieving both lightweighting and performance enhancement.

Response to Reviewer #3

Comment: 1.To make the paper more readable, the code of the proposed algorithm should be open to the readers. Whether thedatabase can be shared.

Response:

Thank you for your suggestion. We have open-sourced the code of the proposed SIS-YOLOv11 algorithm on GitHub, with the access link:

https://github.com/YMW-CC/SIS-YOLOv11.

Comment: 2.A serial of improvements are achieved which are given in the abstract and section of Experimental results, how were these data obtained.

Response:

We appreciate your concern about the validity of the experimental data. We have revised the Abstract to clarify the research design and data sources, and supplemented details in the "Experiment Environments" subsection of the Results section: all comparative models were trained and tested under identical experimental parameters and environmental configurations. This ensures the fairness and reliability of the experimental results, and the data were obtained through standardized training and testing processes based on the above uniform settings.

Comment: 3.Why are the figures not formatted into the main body of the paper.

Response:

Thank you for pointing out this issue. We have re-embedded all figures into the main body of the revised manuscript in the corresponding positions, ensuring that the figures are closely aligned with the text descriptions and improving the readability and coherence of the paper.

Comment: 4.What the meaning of SIS, which appears in SIS-YOLO. The abbreviations in YOLO are not given full spelling or explanation.

Response:

We apologize for the lack of abbreviation explanations. We have supplemented the full name of SIS-YOLOv11 (SimAM-Inception-StyleRandomization (SIS)-YOLOv11) in both the Abstract and Introduction sections. Additionally, we have added the full spelling of YOLO (You Only Look Once) in the Introduction section, ensuring all key abbreviations are clearly explained when first mentioned.

Comment: 5.The authors should emphasize th main contributions and innovations of the d paper, instead of simply introducing the used technologies and database. The main contribution of SIS-YOLOv11.

Response:

Thank you for this constructive comment. We have supplemented relevant content in the Conclusion section to emphasize the core contributions of the paper: the proposed SIS-YOLOv11 framework innovatively integrates four key modules (C3k2-SSI, Fusion-InceptionConv, SPPF-Inception, C2PSA-IS) to address cross-crop generalization and complex climate interference; DepGraph pruning achieves 47.82% parameter reduction while improving performance. The pruned model outperforms baseline and mainstream models, providing a robust lightweight solution for practical cross-crop disease detection.
