# Supplementary material for: A robust cross-crop disease detection framework based on SIS-YOLOv11 with climate-adaptive mechanisms
Source: PLoS One. 2026 Jul 17;21(7):e0353863. doi: 10.1371/journal.pone.0353863 (PMC13378986; doi:10.1371/journal.pone.0353863)
Supplement: Attachment — Submitted filename: Review_Report.docx [file pone.0353863.s001.docx]

# Review Report

Manuscript Title: A Robust Cross-Crop Disease Detection Framework Based on SIS-YOLOv11 with Climate-Adaptive Mechanisms

Manuscript Number: PONE-D-25-35125

## Summary

The manuscript presents a novel cross-crop plant disease detection framework, SIS-YOLOv11, integrating climate-adaptive mechanisms and style randomization to enhance generalization under complex environmental conditions. The work introduces multiple architectural innovations (C3k2-SSI, Fusion-InceptionConv, SPPF-Inception, C2PSA-IS) and employs DepGraph pruning to optimize performance. Experiments on potato and tomato leaf datasets, augmented with simulated haze and rainfall, demonstrate significant improvements in precision, recall, and mAP compared to state-of-the-art models.

## Major Comments

1. Originality and Significance: The study proposes a genuinely novel combination of modules and training strategies tailored for agricultural applications. The focus on cross-crop generalization under adverse climatic noise is highly relevant to real-world agricultural monitoring systems. The integration of style randomization in the feature space for plant disease detection is a noteworthy methodological innovation.
2. Technical Quality and Experimental Design: The architecture modifications are described in sufficient technical detail, with clear diagrams and mathematical formulations. Ablation studies and pruning experiments are well-structured, demonstrating the contribution of each module. Comparisons with multiple strong baselines (YOLO variants, SSD, Faster R-CNN) validate the performance claims. The training environment and hyperparameters are reported, ensuring reproducibility.
3. Data and Methodology Transparency: Dataset composition, augmentation techniques, and experimental setups are described clearly. However, the dataset is not publicly available; it is provided only upon reasonable request. This may limit reproducibility and does not fully meet open-data standards recommended by PLOS ONE.
4. Conclusions and Validity: Conclusions are well supported by the results and visual examples. Limitations are acknowledged, particularly the small dataset size and the focus on two diseases across two crops. The proposed future work directions are appropriate and realistic.

## Minor Comments

5. Some sentences in the introduction are overly long and could be simplified for improved readability.
6. Figures (especially Fig. 5 and Fig. 6) would benefit from clearer legends and slightly larger font sizes in annotations for better visibility.
7. The results section could be enhanced with statistical significance testing to strengthen claims of superiority over baseline models.
8. Clarify whether any data augmentation parameters (e.g., noise intensity for haze/rain) were fixed or randomized during training.

## Ethics and Compliance

Ethical considerations are appropriately addressed; the study involves plant images only, and “N/A” for ethics approval is justified. No competing interests are declared, and no funding sources influenced the work.

## Recommendation

Accept with minor revisions. The manuscript meets high technical and ethical standards, demonstrates originality, and presents results of significant practical relevance. The primary revision needed is to address data availability to improve reproducibility. Minor language and figure clarity improvements are also recommended.

## Specific Suggestions for Revision

9. Upload the dataset (or a substantial portion) to a public repository such as Zenodo, Figshare, or GitHub, and provide the link in the Data Availability Statement.
10. Revise lengthy sentences in the introduction for conciseness.
11. Improve figure clarity by enlarging text and labels in results visualizations.
12. Consider adding statistical tests (e.g., paired t-tests) to support performance differences.
13. Explicitly state whether augmentation parameters were fixed or varied across training runs.
