# Supplementary material for: A robust cross-crop disease detection framework based on SIS-YOLOv11 with climate-adaptive mechanisms
Source: PLoS One. 2026 Jul 17;21(7):e0353863. doi: 10.1371/journal.pone.0353863 (PMC13378986; doi:10.1371/journal.pone.0353863)
Supplement: Peer review history

# Peer review: Decision Letter 0

**DOI:** 10.1371/journal.pone.0353863.r001

**Authors:** Shailender Kumar Verma

23 Nov 2025

PONE-D-25-35125

A Robust Cross-Crop Disease Detection Framework Based on SIS-YOLOv11 with Climate-Adaptive Mechanisms

PLOS ONE

Dear Dr. Qin,

Thank you for submitting your manuscript to PLOS ONE. After careful consideration, we feel that it has merit but does not fully meet PLOS ONE’s publication criteria as it currently stands. Therefore, we invite you to submit a revised version of the manuscript that addresses the points raised during the review process.

Please submit your revised manuscript by Jan 07 2026 11:59PM. If you will need more time than this to complete your revisions, please reply to this message or contact the journal office at plosone@plos.org. When you're ready to submit your revision, log on to https://www.editorialmanager.com/pone/ and select the 'Submissions Needing Revision' folder to locate your manuscript file.

Please include the following items when submitting your revised manuscript:

- A rebuttal letter that responds to each point raised by the academic editor and reviewer(s). You should upload this letter as a separate file labeled 'Response to Reviewers'.
- A marked-up copy of your manuscript that highlights changes made to the original version. You should upload this as a separate file labeled 'Revised Manuscript with Track Changes'.
- An unmarked version of your revised paper without tracked changes. You should upload this as a separate file labeled 'Manuscript'.

If you would like to make changes to your financial disclosure, please include your updated statement in your cover letter. Guidelines for resubmitting your figure files are available below the reviewer comments at the end of this letter.

If applicable, we recommend that you deposit your laboratory protocols in protocols.io to enhance the reproducibility of your results. Protocols.io assigns your protocol its own identifier (DOI) so that it can be cited independently in the future. For instructions see: https://journals.plos.org/plosone/s/submission-guidelines#loc-laboratory-protocols. Additionally, PLOS ONE offers an option for publishing peer-reviewed Lab Protocol articles, which describe protocols hosted on protocols.io. Read more information on sharing protocols at https://plos.org/protocols?utm_medium=editorial-email&utm_source=authorletters&utm_campaign=protocols.

We look forward to receiving your revised manuscript.

Kind regards,

Shailender Kumar Verma, Ph.D.

Academic Editor

PLOS ONE

Journal Requirements:

When submitting your revision, we need you to address these additional requirements.

1. Please ensure that your manuscript meets PLOS ONE's style requirements, including those for file naming. The PLOS ONE style templates can be found at

https://journals.plos.org/plosone/s/file?id=wjVg/PLOSOne_formatting_sample_main_body.pdf and

https://journals.plos.org/plosone/s/file?id=ba62/PLOSOne_formatting_sample_title_authors_affiliations.pdf

2. Please note that PLOS One has specific guidelines on code sharing for submissions in which author-generated code underpins the findings in the manuscript. In these cases, we expect all author-generated code to be made available without restrictions upon publication of the work. Please review our guidelines at https://journals.plos.org/plosone/s/materials-and-software-sharing#loc-sharing-code and ensure that your code is shared in a way that follows best practice and facilitates reproducibility and reuse.

3. We note that you have indicated that there are restrictions to data sharing for this study. PLOS only allows data to be available upon request if there are legal or ethical restrictions on sharing data publicly. For more information on unacceptable data access restrictions, please see http://journals.plos.org/plosone/s/data-availability#loc-unacceptable-data-access-restrictions.

Before we proceed with your manuscript, please address the following prompts:

a) If there are ethical or legal restrictions on sharing a de-identified data set, please explain them in detail (e.g., data contain potentially identifying or sensitive patient information, data are owned by a third-party organization, etc.) and who has imposed them (e.g., a Research Ethics Committee or Institutional Review Board, etc.). Please also provide contact information for a data access committee, ethics committee, or other institutional body to which data requests may be sent.

b) If there are no restrictions, please upload the minimal anonymized data set necessary to replicate your study findings to a stable, public repository and provide us with the relevant URLs, DOIs, or accession numbers. For a list of recommended repositories, please see

https://journals.plos.org/plosone/s/recommended-repositories. You also have the option of uploading the data as Supporting Information files, but we would recommend depositing data directly to a data repository if possible.

We will update your Data Availability statement on your behalf to reflect the information you provide.

4. If the reviewer comments include a recommendation to cite specific previously published works, please review and evaluate these publications to determine whether they are relevant and should be cited. There is no requirement to cite these works unless the editor has indicated otherwise.

[Note: HTML markup is below. Please do not edit.]

Reviewers' comments:

Reviewer's Responses to Questions

**Comments to the Author**

1. Is the manuscript technically sound, and do the data support the conclusions?

The manuscript must describe a technically sound piece of scientific research with data that supports the conclusions. Experiments must have been conducted rigorously, with appropriate controls, replication, and sample sizes. The conclusions must be drawn appropriately based on the data presented.

Reviewer #1: Yes

Reviewer #2: Yes

Reviewer #3: Partly

**********

2. Has the statistical analysis been performed appropriately and rigorously?

Reviewer #1: Yes

Reviewer #2: No

Reviewer #3: No

**********

3. Have the authors made all data underlying the findings in their manuscript fully available?

The PLOS Data policy requires authors to make all data underlying the findings described in their manuscript fully available without restriction, with rare exception (please refer to the Data Availability Statement in the manuscript PDF file). The data should be provided as part of the manuscript or its supporting information, or deposited to a public repository. For example, in addition to summary statistics, the data points behind means, medians and variance measures should be available. If there are restrictions on publicly sharing data—e.g. participant privacy or use of data from a third party—those must be specified.

Reviewer #1: Yes

Reviewer #2: Yes

Reviewer #3: Yes

**********

4. Is the manuscript presented in an intelligible fashion and written in standard English?

PLOS ONE does not copyedit accepted manuscripts, so the language in submitted articles must be clear, correct, and unambiguous. Any typographical or grammatical errors should be corrected at revision, so please note any specific errors here.

Reviewer #1: Yes

Reviewer #2: Yes

Reviewer #3: Yes

**********

5. Review Comments to the Author

Please use the space provided to explain your answers to the questions above. You may also include additional comments for the author, including concerns about dual publication, research ethics, or publication ethics. (Please upload your review as an attachment if it exceeds 20,000 characters)

Reviewer #1: The manuscript presents a novel cross-crop plant disease detection framework, SIS-YOLOv11, integrating climate-adaptive mechanisms and style randomization to enhance generalization under complex environmental conditions. The work introduces multiple architectural innovations (C3k2-SSI, Fusion-InceptionConv, SPPF-Inception, C2PSA-IS) and employs DepGraph pruning to optimize performance. Experiments on potato and tomato leaf datasets, augmented with simulated haze and rainfall, demonstrate significant improvements in precision, recall, and mAP compared to state-of-the-art models.

Reviewer #2: Dear author,

1. The content in the introduction needs improvement.

2. To enhance the model’s generalization and robustness, the authors introduced and modified the Style Randomization module. However, the role of the parameter K within this module is not explained, even though its effect on model accuracy is discussed later. Based on the paper, can K be considered as the weight for randomizing the original data, with K = 1 yielding the best performance on the original samples? If so, what is the significance of analyzing K in the subsequent discussions?

3. In the pruning experiments, the authors did not specify when the pruning operation was performed—was it done before sparse training or before fine-tuning? After pruning, the model’s parameter count decreased by 3.3M, yet all performance metrics improved. Why did the model’s performance increase despite the reduction in parameters? Please provide clarification in the paper.

4. The Discussion section is brief and lacks sufficient depth. The authors are encouraged to expand this section by providing a more thorough analysis of the experimental results, such as explaining why the model's performance improved after pruning.

Reviewer #3: In this manuscript, a novel enhanced YOLOv11n-based object detection algorithm, named SIS-YOLOv11, is proposed for detecting early and late blight potato and tomato leaves under complex backgrounds. This paper needs further improvement to highlight your contributions. The reviewer has the following comments.

1.To make the paper more readable, the code of the proposed algorithm should be open to the readers. Whether thedatabase can be shared.

2.A serial of improvements are achieved which are given in the abstract and section of Experimental results, how were these data obtained.

3.Why are the figures not formatted into the main body of the paper .

4.What the meaning of SIS, which appears in SIS-YOLO. The abbreviations in YOLO are not given full spelling or explanation.

5.The authors should emphasize th main contributions and innovations of the d paper, instead of simply introducing the used technologies and database. The main contribution of SIS-YOLOv11.

**********

6. PLOS authors have the option to publish the peer review history of their article (what does this mean?). If published, this will include your full peer review and any attached files.

If you choose “no”, your identity will remain anonymous but your review may still be made public.

**Do you want your identity to be public for this peer review?**  For information about this choice, including consent withdrawal, please see our Privacy Policy.

Reviewer #1: No

Reviewer #2: No

Reviewer #3: No

**********

[NOTE: If reviewer comments were submitted as an attachment file, they will be attached to this email and accessible via the submission site. Please log into your account, locate the manuscript record, and check for the action link "View Attachments". If this link does not appear, there are no attachment files.]

To ensure your figures meet our technical requirements, please review our figure guidelines: https://journals.plos.org/plosone/s/figures

You may also use PLOS’s free figure tool, NAAS, to help you prepare publication quality figures: https://journals.plos.org/plosone/s/figures#loc-tools-for-figure-preparation.

NAAS will assess whether your figures meet our technical requirements by comparing each figure against our figure specifications.

Attachment Submitted filename: Review_Report.docx

# Author comment: Author response to Decision Letter 1

**DOI:** 10.1371/journal.pone.0353863.r002

11 Apr 2026

Response to Reviewers

Dear Editors and Reviewers,

We sincerely appreciate the valuable comments and constructive suggestions provided by all reviewers. Their insights have greatly helped us improve the quality and completeness of this manuscript. We have carefully addressed each comment and made corresponding revisions to the paper. All modified content in the revised manuscript has been marked in red for the convenience of reviewers to quickly identify. Below is a detailed response to each reviewer’s feedback.

Response to Reviewer #1

Thank you very much for your positive evaluation and recognition of our work. We greatly appreciate your affirmation of the novelty of the SIS-YOLOv11 framework, architectural innovations, and experimental validity.

Response to Reviewer #2

Comment: 1. The content in the introduction needs improvement.

Response:

Thank you for your valuable suggestion. We have comprehensively revised the entire Introduction section to enhance its academic rigor, logical coherence, and readability. Specifically, we optimized the overall structure of the background narrative, supplemented the latest advances in cross-crop disease detection and climate-adaptive modeling, strengthened the comparative analysis between our work and existing literature to highlight the novelty of the proposed framework, and refined the language expression by simplifying cumbersome sentences. The fully revised Introduction section is presented in the revised manuscript.

Comment: 2. To enhance the model’s generalization and robustness, the authors introduced and modified the Style Randomization module. However, the role of the parameter K within this module is not explained, even though its effect on model accuracy is discussed later. Based on the paper, can K be considered as the weight for randomizing the original data, with K = 1 yielding the best performance on the original samples? If so, what is the significance of analyzing K in the subsequent discussions?

Response:

We thank the reviewer for this valuable comment. We have added detailed explanations of parameter K in the Style Randomization module section of the revised paper. K denotes the number of domain-specific sub-modules in the module: K=0 represents no style randomization; increasing K enables the model to learn style variations across more crop domains, while an unsuitable K (either too large or too small) will lead to overfitting or insufficient generalization respectively. The adjustable K allows flexible adaptation to different cross-crop detection tasks, which is a core merit of our module. The subsequent analysis of K is thus to quantitatively validate the effect of this parameter on the model’s generalization and robustness, and further confirm the superiority of our adjustable design.

Comment: 3. In the pruning experiments, the authors did not specify when the pruning operation was performed—was it done before sparse training or before fine-tuning? After pruning, the model’s parameter count decreased by 3.3M, yet all performance metrics improved. Why did the model’s performance increase despite the reduction in parameters? Please provide clarification in the paper.

Response:

We appreciate your observation and supplemented details in the DepGraph Pruning section: (1) Pruning is implemented prior to sparse training to eliminate redundant structures; (2) Fine-tuning is exclusively conducted on the pruned lightweight model. Performance improvement after parameter reduction is attributed to pruning-removed feature interference and fine-tuning-optimized parameter distribution, achieving both lightweighting and performance enhancement.

Comment: 4. The Discussion section is brief and lacks sufficient depth. The authors are encouraged to expand this section by providing a more thorough analysis of the experimental results, such as explaining why the model's performance improved after pruning.

Response:

We appreciate your observation and supplemented details in the DepGraph Pruning section: (1) Pruning is implemented prior to sparse training to eliminate redundant structures; (2) Fine-tuning is exclusively conducted on the pruned lightweight model. Performance improvement after parameter reduction is attributed to pruning-removed feature interference and fine-tuning-optimized parameter distribution, achieving both lightweighting and performance enhancement.

Response to Reviewer #3

Comment: 1.To make the paper more readable, the code of the proposed algorithm should be open to the readers. Whether thedatabase can be shared.

Response:

Thank you for your suggestion. We have open-sourced the code of the proposed SIS-YOLOv11 algorithm on GitHub, with the access link:

https://github.com/YMW-CC/SIS-YOLOv11.

Comment: 2.A serial of improvements are achieved which are given in the abstract and section of Experimental results, how were these data obtained.

Response:

We appreciate your concern about the validity of the experimental data. We have revised the Abstract to clarify the research design and data sources, and supplemented details in the "Experiment Environments" subsection of the Results section: all comparative models were trained and tested under identical experimental parameters and environmental configurations. This ensures the fairness and reliability of the experimental results, and the data were obtained through standardized training and testing processes based on the above uniform settings.

Comment: 3.Why are the figures not formatted into the main body of the paper.

Response:

Thank you for pointing out this issue. We have re-embedded all figures into the main body of the revised manuscript in the corresponding positions, ensuring that the figures are closely aligned with the text descriptions and improving the readability and coherence of the paper.

Comment: 4.What the meaning of SIS, which appears in SIS-YOLO. The abbreviations in YOLO are not given full spelling or explanation.

Response:

We apologize for the lack of abbreviation explanations. We have supplemented the full name of SIS-YOLOv11 (SimAM-Inception-StyleRandomization (SIS)-YOLOv11) in both the Abstract and Introduction sections. Additionally, we have added the full spelling of YOLO (You Only Look Once) in the Introduction section, ensuring all key abbreviations are clearly explained when first mentioned.

Comment: 5.The authors should emphasize th main contributions and innovations of the d paper, instead of simply introducing the used technologies and database. The main contribution of SIS-YOLOv11.

Response:

Thank you for this constructive comment. We have supplemented relevant content in the Conclusion section to emphasize the core contributions of the paper: the proposed SIS-YOLOv11 framework innovatively integrates four key modules (C3k2-SSI, Fusion-InceptionConv, SPPF-Inception, C2PSA-IS) to address cross-crop generalization and complex climate interference; DepGraph pruning achieves 47.82% parameter reduction while improving performance. The pruned model outperforms baseline and mainstream models, providing a robust lightweight solution for practical cross-crop disease detection.

Attachment Submitted filename: Response to Reviewers.pdf

# Peer review: Decision Letter 1

**DOI:** 10.1371/journal.pone.0353863.r003

**Authors:** Shailender Kumar Verma

1 Jul 2026

A Robust Cross-Crop Disease Detection Framework Based on SIS-YOLOv11 with Climate-Adaptive Mechanisms

PONE-D-25-35125R1

Dear Dr. Qin,

We’re pleased to inform you that your manuscript has been judged scientifically suitable for publication and will be formally accepted for publication once it meets all outstanding technical requirements.

Within one week, you’ll receive an e-mail detailing the required amendments. When these have been addressed, you’ll receive a formal acceptance letter and your manuscript will be scheduled for publication.

An invoice will be generated when your article is formally accepted. Please note, if your institution has a publishing partnership with PLOS and your article meets the relevant criteria, all or part of your publication costs will be covered. Please make sure your user information is up-to-date by logging into Editorial Manager at Editorial Manager® and clicking the ‘Update My Information' link at the top of the page. For questions related to billing, please contact billing support.

If your institution or institutions have a press office, please notify them about your upcoming paper to help maximize its impact. If they’ll be preparing press materials, please inform our press team as soon as possible -- no later than 48 hours after receiving the formal acceptance. Your manuscript will remain under strict press embargo until 2 pm Eastern Time on the date of publication. For more information, please contact onepress@plos.org.

Kind regards,

Chetan Keswani, Ph.D.

Academic Editor

PLOS One

Additional Editor Comments (optional):

Reviewers' comments:

Reviewer's Responses to Questions

**Comments to the Author**

1. If the authors have adequately addressed your comments raised in a previous round of review and you feel that this manuscript is now acceptable for publication, you may indicate that here to bypass the “Comments to the Author” section, enter your conflict of interest statement in the “Confidential to Editor” section, and submit your "Accept" recommendation.

Reviewer #2: All comments have been addressed

Reviewer #3: All comments have been addressed

**********

2. Is the manuscript technically sound, and do the data support the conclusions?

The manuscript must describe a technically sound piece of scientific research with data that supports the conclusions. Experiments must have been conducted rigorously, with appropriate controls, replication, and sample sizes. The conclusions must be drawn appropriately based on the data presented.

Reviewer #2: Yes

Reviewer #3: Yes

**********

3. Has the statistical analysis been performed appropriately and rigorously?

Reviewer #2: Yes

Reviewer #3: Yes

**********

4. Have the authors made all data underlying the findings in their manuscript fully available?

The PLOS Data policy requires authors to make all data underlying the findings described in their manuscript fully available without restriction, with rare exception (please refer to the Data Availability Statement in the manuscript PDF file). The data should be provided as part of the manuscript or its supporting information, or deposited to a public repository. For example, in addition to summary statistics, the data points behind means, medians and variance measures should be available. If there are restrictions on publicly sharing data—e.g. participant privacy or use of data from a third party—those must be specified.

Reviewer #2: Yes

Reviewer #3: Yes

**********

5. Is the manuscript presented in an intelligible fashion and written in standard English?

PLOS ONE does not copyedit accepted manuscripts, so the language in submitted articles must be clear, correct, and unambiguous. Any typographical or grammatical errors should be corrected at revision, so please note any specific errors here.

Reviewer #2: Yes

Reviewer #3: Yes

**********

6. Review Comments to the Author

Please use the space provided to explain your answers to the questions above. You may also include additional comments for the author, including concerns about dual publication, research ethics, or publication ethics. (Please upload your review as an attachment if it exceeds 20,000 characters)

Reviewer #2: (No Response)

Reviewer #3: The authors have addressed all the comments. The paper is recommended to be accepted after a minor revision.

**********

7. PLOS authors have the option to publish the peer review history of their article (what does this mean?). If published, this will include your full peer review and any attached files.

If you choose “no”, your identity will remain anonymous but your review may still be made public.

**Do you want your identity to be public for this peer review?**  For information about this choice, including consent withdrawal, please see our Privacy Policy.

Reviewer #2: No

Reviewer #3: No

**********

# Editor report: Acceptance letter

**DOI:** 10.1371/journal.pone.0353863.r004

**Authors:** Chetan Keswani

PONE-D-25-35125R1

PLOS One

Dear Dr. Qin,

I'm pleased to inform you that your manuscript has been deemed suitable for publication in PLOS One. Congratulations! Your manuscript is now being handed over to our production team.

At this stage, our production department will prepare your paper for publication. This includes ensuring the following:

* All references, tables, and figures are properly cited

* All relevant supporting information is included in the manuscript submission,

* There are no issues that prevent the paper from being properly typeset

You will receive further instructions from the production team, including instructions on how to review your proof when it is ready. Please keep in mind that we are working through a large volume of accepted articles, so please give us a few days to review your paper and let you know the next and final steps.

Lastly, if your institution or institutions have a press office, please let them know about your upcoming paper now to help maximize its impact. If they'll be preparing press materials, please inform our press team within the next 48 hours. Your manuscript will remain under strict press embargo until 2 pm Eastern Time on the date of publication. For more information, please contact onepress@plos.org.

You will receive an invoice from PLOS for your publication fee after your manuscript has reached the completed accept phase. If you receive an email requesting payment before acceptance or for any other service, this may be a phishing scheme. Learn how to identify phishing emails and protect your accounts at https://explore.plos.org/phishing.

If we can help with anything else, please email us at customercare@plos.org.

Thank you for submitting your work to PLOS ONE and supporting open access.

Kind regards,

PLOS ONE Editorial Office Staff

on behalf of

Dr. Chetan Keswani

Academic Editor

PLOS One
